# Therapeutic effect and safety of Tuina on sciatica: A protocol for systematic review and meta-analysis^∗^

**DOI:** 10.1097/MD.0000000000028097

**Published:** 2021-12-03

**Authors:** Huixin Yan, Yun An, Tao Zhang, Jiangna Zhao, Juntao Yan

**Affiliations:** Department of Tuina, Yueyang Hospital of Integrated Traditional Chinese and Western Medicine, Shanghai University of Traditional Chinese Medicine, Shanghai, PR China.

**Keywords:** meta-analysis, protocol, sciatica, systematic review, Tuina

## Abstract

**Background:**

Sciatica is one of the common pain symptoms in the human body, also known as radiating leg pain. Sciatica is increasingly occurring due to poor posture and lack of physical exercise all over the world. At present, many studies have indicated that Tuina can improve the clinical symptoms and functional status of sciatica. However, there is currently no relevant systematic review to evaluate and report this clinical scientific issue. Consequently, this study will conduct a meta-analysis on the effectiveness and safety of Tuina therapy for sciatica.

**Methods and analysis:**

Randomized controlled trials (RCTs) related to Tuina treatment of sciatica will be retrieved from the Chinese and English databases and Clinical Trial Register. These databases include China National Knowledge Infrastructure, Wan Fang Database, Chinese Biomedical Literature Database, VIP Database for Chinese Technical Periodicals, PubMed, Embase, Web of Science, Cochrane Library, and Medline, etc. We will consider articles published in English or Chinese between database initiation and October 2021. Our team will use Review Manager Software 5.3 software provided by the Cochrane Collaborative Network to conduct this systematic review and meta-analysis.

**Results:**

This study provides a comprehensive evaluation of the effectiveness and safety of Tuina therapy for sciatica.

**Conclusion:**

The conclusion of our study will provide scientific evidence and reference to determine whether Tuina is an effective and safe intervention for patients with sciatica.

**Registration number:**

INPLASY2021100034.

## 1 Introduction

The International Association for the Study of Pain revised the definition of “pain” in 2020 to “An unpleasant sensory and emotional experience associated with, or resembling that associated with, actual or potential tissue damage.”^[1]^ However, the sciatic nerve is the largest nerve in the human body, and one of the most common clinical pain symptoms is sciatica.^[2,3]^ Previous studies have shown that the prevalence of sciatica ranges from 1.2% to 43%.^[4]^ Sciatica is a clinical syndrome with symptoms of pain or paresthesia along the distribution of the sciatic nerve or in the associated lumbosacral nerve roots.^[5,6]^ It can be divided into two types: primary sciatica and secondary sciatica. Epidemiological studies have revealed that there are many factors affecting the onset of sciatica, such as age, sex, occupation, parity, body habitus, genetic factors, environmental factors, and so on. ^[7]^ Conservative management, exercise therapy, manual therapy, medication, spinal injections, surgical management, postoperative physiotherapy and other methods are all treatment methods for sciatica. Up to now, conservative treatment is the initial and preferred method, which is mainly to reduce pressure and inflammation to control pain and maintain function. Clinically, the treatment of sciatica with analgesic drugs and nerve-nourishing drugs (analgesics, sedatives, vasodilators and B vitamins, etc.) is not satisfactory. ^[8–10]^

Tuina, also known as massage, is a complementary and alternative therapy. It has been used to relieve pain symptoms caused by various diseases for thousands of years. Some scholars and experts believe that pain has become the “the fifth vital sign.” ^[11]^ Studies have indicated that Tuina or massage can effectively relieve the pain and functional limitation symptoms of sciatica.^[12–14]^ In many randomized controlled trial (RCT) studies and Tuina textbooks, doctors and teachers introduced Tuina therapy for the treatment of sciatica, such as Huantiao (GB 30), Yinmen (BL 37), Weizhong (BL 40), Chengshan (BL 57), Shenshu (BL 23), and Dachangshu (BL 23), etc.^[15–17]^ However, there is still no published review of evidence or protocols on the efficacy and safety of massage in the treatment of sciatica. In order to fill the gap in this scientific question, our team will conduct related systematic reviews and meta-analysis.

## 2 Methods and analysis

### 2.1 Study registration

Our team's research will be performed by adhering to the guidelines of the preferred reporting items for systematic review and meta-analysis protocols (PRISMA-P) 2015.^[18]^ The study was registered at the International Platform of Registered Systematic Review and Meta-Analysis Protocols (INPLASY) on October 10, 2021 (registration number INPLASY2021100034).

### 2.2 Inclusion criteria

#### 2.2.1 Types of participants

Our study will include patients of any age group who have been diagnosed with sciatica. Furthermore, the disease will include primary sciatica and secondary sciatica. No limitations of nationality, age, gender, profession, location, ethnicity, educational background, and economic status were imposed. However, studies on patients with serious diseases such as lumbar vertebral tumors, lumbar fractures, pelvic tumors, rheumatoid arthritis, ankylosing spondylitis, cardiovascular diseases, and mental diseases will be excluded.

#### 2.2.2 Types of interventions

Simple Tuina therapy or Tuina therapy combined with other ordinary and basic treatment will be the intervention of the experimental group. Nevertheless, we will not limit the types of Tuina techniques, treatment acupoints and meridians, treatment time, treatment frequency and other factors. If the control group is treated with Chinese herbal medication, placebo, western medication, acupuncture, acupoint injection, traction and so on, or even with no treatment, will be included. In short, Tuina therapy should be the only difference between the experimental group and the control group.

#### 2.2.3 Types of studies

Only RCTs using Tuina to relieve pain symptoms and related dysfunction in patients with sciatica will be included. The language of articles included by our team will be limited to Chinese and English. The studies involving non-RCTs, animal mechanism experiments, retrospective studies, case reports, cohort studies, and review articles will be excluded.

#### 2.2.4 Types of outcomes

The main outcomes of this review are clinical effective rate and visual analogue scale. The additional outcomes include pain threshold, Oswestry Disability Index (ODI), modified Japanese Orthopaedic Association (JOA) score, six-point behavior (BRS-6) score, six-point behavior (BRS-6) score, adverse reactions, etc.

### 2.3 Data sources and search methods

#### 2.3.1 Electronic searches

RCTs related to Tuina treatment of sciatica will be retrieved from the Chinese and English databases. These databases include China National Knowledge Infrastructure, Wan Fang Database, Chinese Biomedical Literature Database, VIP Database for Chinese Technical Periodicals, PubMed, Embase, Web of Science, Cochrane Library, and Medline, etc. We will consider articles published in English or Chinese between database initiation and October 2021. According to the “PICOS” (patients, intervention, comparison, outcome, and study design) criteria, we use the following search terms: sciatica, sciatic neuralgia, bilateral sciaticas, Tuina, massage, manipulation, randomized controlled trial, RCT, etc. Our team's search strategy for PubMed is presented in Table 1. The specific search strategy was adjusted according to the characteristics of different databases.

**Table 1:** Search strategy for the PubMed database.

Number	Search items
1	Tuina
2	Massage
3	Manipulation
4	Zone Therapy
5	Manual therapy
6	Massotherapy
7	Acupressure
8	Osteopathic Manipulation
9	1 OR 2–8
10	Sciatica
11	Sciatic Neuralgia
12	Neuralgia, Sciatic
13	Neuralgias, Sciatic
14	Sciatic Neuralgias
15	Sciatica, Bilateral
16	Bilateral Sciatica
17	Bilateral Sciaticas
18	10 OR 11-17
19	Randomized controlled trial
20	Controlled clinical trial
21	Clinical trial
22	Randomly
23	Placebo
24	Trial
25	RCT
26	19 OR 20-25
27	9 And 18 And 26

#### 2.3.2 Searching for other resources

Team members will also use related search terms to obtain in the Chinese Clinical Registry and the International Clinical Trials Registry Platform (ICTRP). In addition, we will manually search for the original literature to find possible related trials, and tried to obtain gray literature from other sources.

### 2.4 Data selection

**Figure 1:**
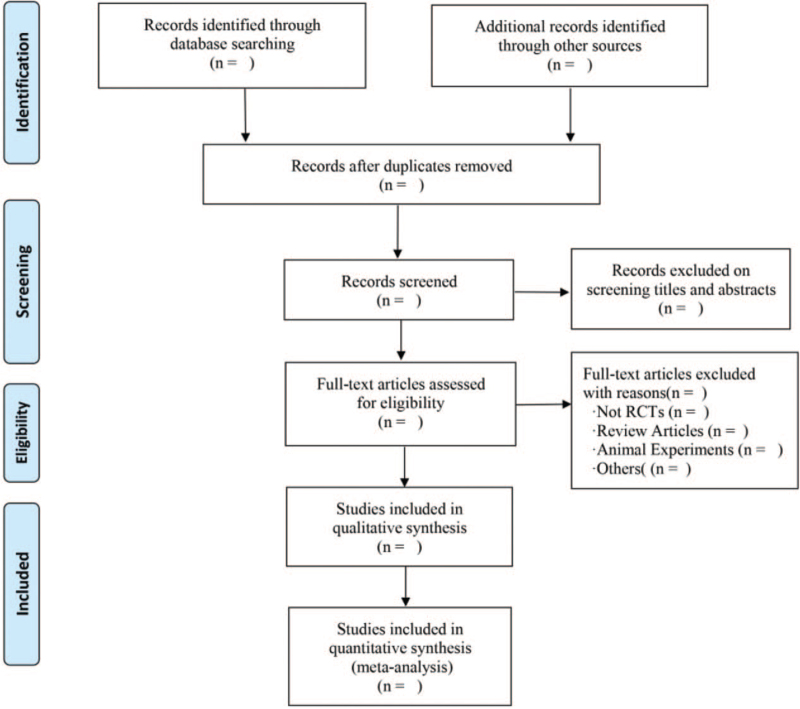
Flow diagram of the study selection process.

Before the start of the study, all authors will carefully study the Preferred Reporting Items for Systematic Reviews and Meta-Analyses and Cochrane Handbook for Systematic Reviews of Interventions. In order to ensure the reliability and scientificity of the screening, we will use NoteExpress V3.0 software and Endnote X9.1 software to manage articles. According to the previously established inclusion and exclusion criteria, the two researchers will conduct preliminary screening, re-screening and in-depth review of the literature. First of all, we will use the management software to delete duplicate records to complete the preliminary screening. The two authors will complete the re-screening by reading the titles, abstracts and keywords of these articles. Finally, we will read the full text of the remaining literature and conduct an in-depth review to further determine whether to include or exclude. If there are any differences throughout the process, the researchers will discuss, negotiate and resolve them. The flow chart of literature search and screening process will be showed in Figure 1.

### 2.5 Data extraction and analysis

According to the inclusion and exclusion criteria, our research team will first make a data extraction table for this study. The basic information we need to extract for the final included study will include: first author, publication year, article title, trial location, relevant information about the study population, research methodology information, intervention measures, treatment time, treatment frequency, primary and secondary Information on outcome indicators and adverse reactions. In the process of collecting information, any disagreements will be resolved through team negotiation. If significant information and content is missing, we will try to contact the respective corresponding author using e-mail.

### 2.6 Risk of bias assessment

The researchers of our team will use the Cochrane Collaborative tool to independently assess the risk of bias in all final inclusion of trials. In each study, seven items (Random sequence generation, allocation concealment, blinding of participants and personnel, blindness of outcome assessments, incomplete outcome data, selective reporting, and other bias) will be evaluated as unclear, high, or low risk. The results of the bias assessment risk will be carefully checked by other researchers, and any disagreement will be finalized after further discussion by all investigators.

### 2.7 Data synthesis and analysis

Review Manager software (Revman, Version 5.3 for Windows) will be used to perform the meta- analysis. The Chi-Squared test and *I*^2^ statistic will be used to assess the heterogeneity of literature according to the values of *P* and *I*^2^. If the homogeneity is low (*P* > .1; *I*^2^ < 50%), the fixed-effect model will be used for the meta-analysis. When there is high heterogeneity, we will first try to find the reason for it, and then decide the meta-analysis model to be finally selected. If the specific cause cannot be found and the heterogeneity is within an acceptable range, we will finally choose the random effect model for meta-analysis.

### 2.8 Assessment of reporting biases

If the number of included studies exceeds 10 trials, we will use Review Manager Software 5.3 software to generate funnel plots to report publication bias. When our team discovers an asymmetric funnel chart, the researchers will try to use Egger and Begg test to assess potential publication bias.

### 2.9 Subgroup analysis

Our team firstly will consider the subgroup analysis of primary sciatica and secondary sciatica to explore the influence of massage on different types of sciatica. If conditions permit, we will also consider subgroup analysis from the following factors such as the types of Tuina techniques, treatment acupoints and meridians, treatment time, intervention frequency, age, gender, region, sample size, etc.

### 2.10 Sensitivity analysis

During the test review process, the sensitivity analysis will review the robustness of the main decisions. After excluding individual low-quality studies, our team will conduct a meta-analysis again to assess the quality and robustness of the conclusions, compare the results and initiate discussions.

### 2.11 Grading the quality of evidence

According to the Grading of Recommendations Assessment, Development and Evaluation (GRADE) system, ^[19]^ 2 investigators will independently assess the quality of evidence. Based on the five rating standards (limitation, indirectness, inaccuracy, inconsistency, and publication bias), the quality of evidence will be divided into 4 levels (very low, low, medium, and high). ^[20]^

### 2.12 Ethics and dissemination

Since our study is a meta-analysis of existing RCT literature and does not involve any personal privacy, the agreement does not require ethical approval. In addition, we will publish the results in peer-reviewed journals to evaluate the effectiveness and safety of Tuina treatment for sciatica.

## 3 Discussion

Sciatica belongs to the category of “arthralgia syndrome” or “low-back and leg pain.” Chinese clinicians believe that after trauma or exogenous wind, cold, dampness, and/or heat pathogens, qi stagnation and blood stasis in the meridians and collaterals will lead to sciatica. According to the theory of meridians and acupoints in traditional Chinese medicine, its disease location is in the bladder channel of foot taiyang and the gallbladder channel of foot shaoyang. Tuina is one of the characteristic external therapies of traditional Chinese medicine. The main mechanism of Tuina to treat pain is that Tuina is operated on the meridians, acupoints or specific parts of the human body, which can regulate yin-yang balance, dredge the meridians collaterals, run qi and blood, nourish the muscles and bones, thereby improving the clinical symptoms such as pain and limited mobility. From the perspective of modern medicine, Tuina therapy can expand capillaries, accelerate blood flow, improve the microcirculation of the body, reduce the accumulation of local pain factors, and achieve the purpose of alleviating or eliminating pain.^[21]^ In short, Tuina has the characteristics and advantages of being relatively safe and comfortable, less side effects, multi-channel, and multi-target, and it is deeply loved and welcomed by patients.^[22]^

Many previous studies have shown that Tuina helps to improve the clinical symptoms of pain and functional limitation in patients with sciatica. However, no systematic evaluation and meta-analysis of the clinical efficacy and safety of Tuina therapy for sciatica have been published in China and abroad. Therefore, this study is the first systematic study of Tuina therapy for sciatica, in order to provide some reference for clinicians and physiotherapists to treat this disease. Meanwhile, our team hopes to further promote the development and promotion of Tuina therapy for pain.

## Author contributions

**Conceptualization:** Juntao Yan.

**Data curation:** Yun An, Tao Zhang, Jiangna Zhao.

**Formal analysis:** Huixin Yan.

**Funding acquisition:** Juntao Yan.

**Project administration:** Juntao Yan.

**Validation:** Huixin Yan, Tao Zhang.

**Writing – original draft:** Huixin Yan, Yun An, Tao Zhang, Jiangna Zhao.

**Writing – review & editing:** Huixin Yan, Yun An, Tao Zhang.
